# Sex-related disparities in cognitive impairment in kidney transplant patients with kidney failure

**DOI:** 10.1007/s40620-025-02436-w

**Published:** 2025-10-25

**Authors:** Piotr Olejnik, Aleksandra Golenia, Oliwia Maciejewska, Dominika Kurzawa, Ewa Wojtaszek, Jolanta Małyszko

**Affiliations:** 1 Department of Neurology, Medical University of Warsaw, 02-097 Warsaw, Poland; 2 Doctoral School, Medical University of Warsaw, 02-093 Warsaw, Poland; 3 Department of Neurology, University Clinical Center, Medical University of Warsaw, 02-097 Warsaw, Poland; 4 Department of Nephrology, Dialysis and Internal Medicine, Medical University of Warsaw, 02-097 Warsaw, Poland

**Keywords:** Cognitive impairment, Sex-related disparities, Kidney failure, Kidney transplantation

## Abstract

**Background:**

There are unexplained sex differences regarding prevalence, morbidity, and mortality in chronic kidney disease (CKD). Of note, females are less likely to be waitlisted and experience longer waiting times for kidney transplant (KTx). Recently, interest in cognitive impairment among CKD patients has increased due to its potential negative impact on therapeutic outcomes. This study aimed to investigate the influence of sex on cognitive impairment prevalence and patterns, as well as modifiable dementia risk factors, in KTx recipients.

**Methods:**

This cross-sectional study screened KTx recipients for cognitive impairment using the Mini-Addenbrooke’s Cognitive Examination. Demographic data, medical histories, and laboratory results were collected from medical records.

**Results:**

The study included 126 consecutive KTx recipients, predominantly male (62%). Males showed higher serum creatinine (1.57 vs. 1.25 mg/dL; *p* = 0.001), urea (59.50 vs. 55.00 mg/dL; *p* = 0.046), and uric acid (7.20 vs. 6.20 mg/dL; *p* = 0.012) levels compared to females. However, creatinine clearance rates did not differ significantly between sexes (49.03 ± 20.29 vs. 50.79 ± 21.7 mL/min/1.73 m^2^; *p* = 0.645). A non-significant trend toward higher smoking prevalence among males was observed (*p* = 0.054). Cognitive impairment prevalence in the overall cohort was 23%, with no significant sex difference (males: 23.1%, females: 22.9%).

**Conclusions:**

There was a high prevalence of cognitive impairment among KTx recipients, affecting both sexes equally. Male predominance in the study cohort likely reflects systemic disparities in transplant access. These findings highlight the importance of integrating sex-specific considerations into the management and follow-up care of KTx recipients.

**Graphical abstract:**

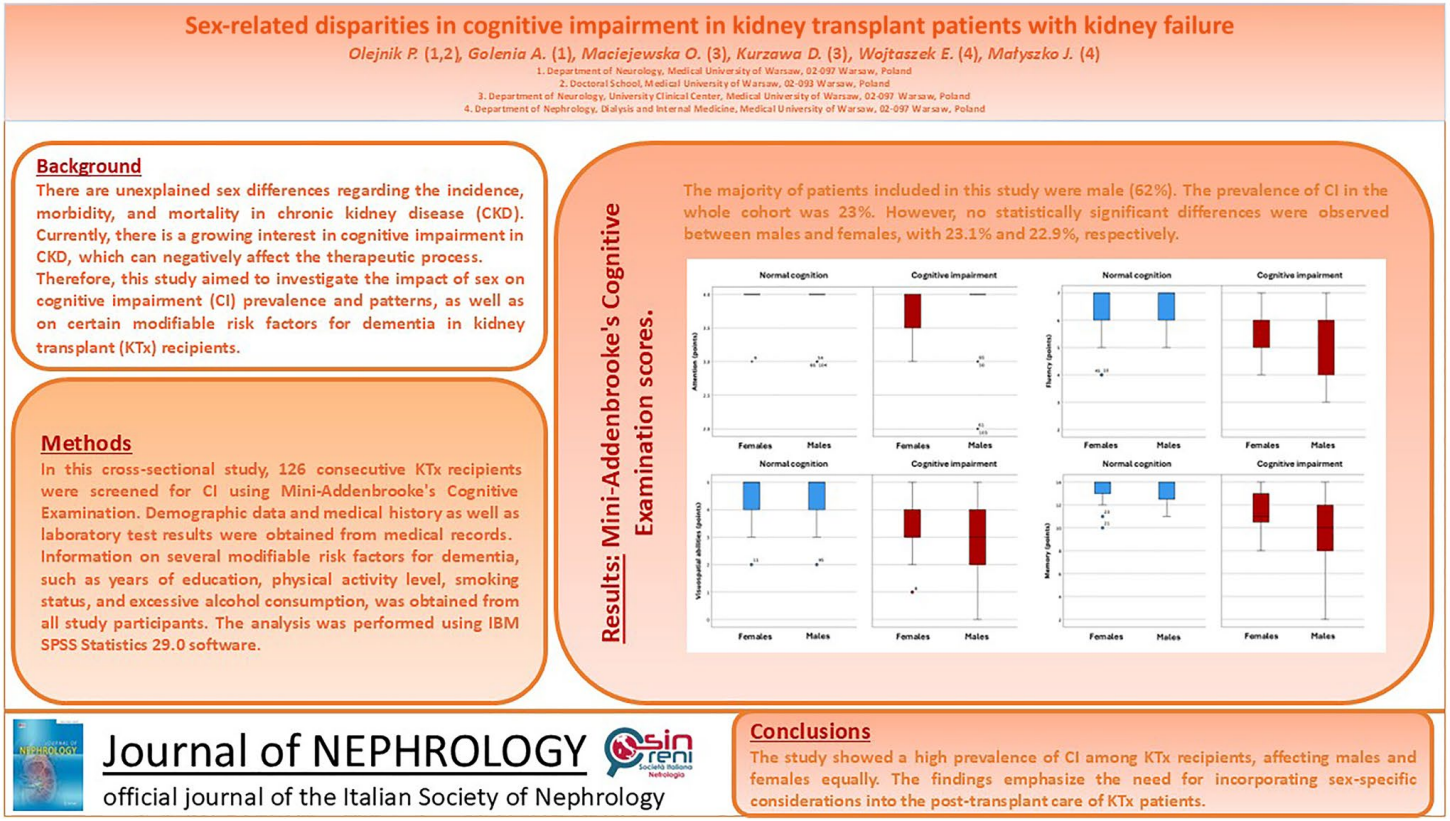

## Introduction

The prevalence of chronic kidney disease (CKD) has risen dramatically in recent decades, partially due to an increase in its most common causes such as diabetes mellitus, hypertension, and aging [1]. CKD is currently the 10th leading cause of death globally and the 8th leading cause of death in high-income countries [1].

Unexplained sex differences exist regarding the incidence, morbidity, and mortality in various CKD stages, progression to kidney failure, and initiation of kidney replacement therapy (KRT) [2]. In 2017, the prevalence of CKD stages G1 to G3 was 1.29 times higher in females than in males [2]. However, an analysis of data from six outpatient clinics in Austria, found that more males than females receive nephrology care [3]. Additionally, males show higher incidence rates of newly initiated KRT and higher mortality, suggesting a more rapid progression to kidney failure [2]. Females are less likely to be waitlisted and must wait longer for a kidney transplant (KTx), which is the preferred treatment for patients with kidney failure [4]. It seems that the role of sex in the progression of kidney disease in humans remains unclear and much more complex than previously thought. Interactions between various biological and non-biological factors should be considered [5].

Currently, there is a growing interest in cognitive disorders in both CKD and kidney failure, which can negatively affect the therapeutic process [6]. As previously demonstrated, cognitive impairment is highly prevalent in patients undergoing various forms of KRT, with the highest rates observed in hemodialysis patients in comparison with peritoneal dialysis patients and KTx recipients [7–9]. On the other hand, little is known about sex-specific disparities regarding the prevalence of health and social factors affecting cognition in kidney failure. However, it is well documented that females have a higher prevalence of Alzheimer’s disease than males, not only because females live longer, but also due to variable modifiable risk factors or biological influences, such as hormonal changes (e.g., estrogen decline after menopause), sex-specific genetic expression, and differences in brain structure and function [10]. Preclinical studies support this thesis, indicating that female triple transgenic mice, an animal model of Alzheimer’s disease, show significantly higher β-amyloid burden and greater behavioral deficits than age-matched male mice [11]. Therefore, it seems that sex steroid hormones contribute to cognitive impairment development. Also, males and females tend to perform better on different cognitive tasks [10]. For instance, males tend to do better on tasks that require mental rotation and visuospatial working memory, while females perform better on tasks that require verbal abilities, such as phonemic and semantic fluency, which reflect executive functions, as well as location memory tasks [10]. While some patients with kidney failure undergoing dialysis exhibit executive dysfunction despite showing no general cognitive impairment on the Mini-Mental State Examination [12], they might differ based on sex-related cognitive aptitude as well.

Finally, modifiable risk factors that relate to lifestyle and behavior can increase or decrease the risk of dementia [13]. There are several known, modifiable risk factors, such as lower education, hypertension, diabetes mellitus, depression, insufficient physical activity, social isolation, smoking, or excessive alcohol consumption [13].

Therefore, the aim of the present study was to investigate the impact of sex on: (i) cognitive impairment prevalence, (ii) cognitive impairment patterns, and (iii) certain modifiable risk factors for dementia in patients with kidney failure after KTx.

## Materials and methods

This cross-sectional study included adult KTx recipients. The study participants were enrolled between 1st August, 2022 and 12th February, 2025 during routine visits at the transplant outpatient clinic of the Medical University of Warsaw, subsequent to obtaining informed consent. Cognitive function was assessed once, at the time of study enrollment, at least 4 weeks following KTx. Subjects were enrolled only if they had a history of kidney failure and had undergone KTx, were 18 years of age or older, were Polish speakers, and had consulted with their treatment team. Only clinically stable patients without infectious diseases in the previous 8 weeks, decompensated heart, liver failure, psychiatric or neurodegenerative disorders, or delirium were enrolled in the study. Demographic data (e.g. age, sex) and medical history (e.g. primary renal disease, duration of dialysis, comorbidities, and results of laboratory tests) were obtained from medical records as part of the routine standard of care. Information on several modifiable risk factors for dementia, such as years of education, physical activity level, smoking status, and excessive alcohol consumption, was obtained from all study participants. A detailed description of how the data regarding modifiable risk factors for dementia were collected is described elsewhere [13]. Exclusion criteria included a language barrier, physical disabilities such as vision impairment or limb paresis. Also, participants with a previously documented diagnosis of severe cognitive impairment or dementia were excluded to ensure capacity to consent. Mild or undiagnosed cognitive impairment was not an exclusion criterion.

The local Ethics Committee approved the study protocol (approval number KB/81/2022), and informed consent was obtained from all participants. The study adhered to the ethical guidelines of the World Medical Association’s Declaration of Helsinki.

### Cognitive function assessment

Cognitive functions were evaluated using the Mini-Addenbrooke’s Cognitive Examination (MACE), a brief and sensitive screening battery, derived from the Addenbrooke’s Cognitive Examination III [14]. The MACE takes approximately 5–7 min to complete and assesses four cognitive domains: orientation to time, memory (learning and recalling 7-item name and address), language (as semantic fluency), and visuospatial abilities (a clock drawing task). The final score is the sum of the subscales and ranges from 0 to 30 points [14]. Although the MACE is a relatively new tool that has not yet been analyzed in many studies, it has been shown to distinguish patients with dementia [14, 15]. According to Hsieh and co-workers, MACE scores of 25 points or lower indicate potential cognitive impairment [14].

### Statistical analysis

The analysis was performed using IBM SPSS Statistics 29.0 software. The Shapiro–Wilk test was used to assess the normality of data distribution. Student’s t-test was applied for continuous variables which followed normal distribution, while a non-parametric test, the Mann–Whitney U test, was used to test for differences in continuous parameters with non-normal distribution. The Chi-squared test of independence was used to compare categorical variables between the groups. Additionally, the one-sample Chi-squared test was employed to assess the significance of deviations between observed and expected frequencies. A *p-*value < 0.05 was considered statistically significant.

## Results

### Baseline demographic and clinical characteristics of the study population

This study included 126 consecutive patients with kidney failure who underwent KTx, the majority of whom were male (62%). Table 1 summarizes the demographic and laboratory test results, as well as the comparison of selected modifiable risk factors for dementia between the study groups.

**Table 1:** Demographic characteristics of the study population

	All participants	Females	Males	*p*-value
General characteristics				
Number, *n (%)*	126 (100)	48 (38.1)	78 (61.9)	0.008^a,*^
Age, years, *mean* ± *SD*	51.20 ± 12.67	50.77 ± 12.92	51.46 ± 12.59	0.768^b^
Cognitive impairment prevalence, *n (%)*	29 (23)	11 (22.9)	18 (23.1)	0.983^c^
Time from KTx to cognitive assessment, months, *median* *[Q1; Q3]*	2.49 [1.17; 11.18]	2.45 [0.94; 11.84]	2.54 [1.39; 11.18]	0.274^d^
Dialysis vintage, months, *median* *[Q1; Q3]*	24.00 [14.00; 36.00]	24.00 [14.25; 43.50]	23.50 [13.75; 36.00]	0.788^d^
Charlson Comorbidity Index, *median* *[Q1; Q3]*	3.00 [2.00; 4.00]	3.00 [2.25; 4.00]	3.00 [2.00; 5.00]	0.928^d^
Biochemical data				
Creatinine level (mg/dl), *median* *[Q1; Q3]*	1.46 [1.19; 1.93]	1.25 [1.00; 1.65]	1.57 [1.30; 2.18]	0.001^d,*^
Creatinine clearance (ml/min/1,73m^2^), *mean* ± *SD*	49.70 ± 20.77	50.79 ± 21.70	49.03 ± 20.29	0.645^b^
Urea level (mg/dl), *median* *[Q1; Q3]*	58.00 [44.75; 79.00]	55.00 [35.00; 69.25]	59.50 [46.75; 84.75]	0.046^d,*^
Uric acid level (mg/dl), *median* *[Q1; Q3]*	6.90 [5.80; 8.30]	6.20 [5.25; 7.60]	7.20 [6.10; 8.63]	0.012^d,*^
Hemoglobin level (g/dl), *median* *[Q1; Q3]*	11.50 [10.48; 13.13]	11.45 [10.43; 12.68]	11.70 [10.48; 13.50]	0.325^d^
Tacrolimus level (ng/ml), *median* *[Q1; Q3]*	13.15 [9.60; 16.60]	12.80 [10.23; 16.60]	13.35 [9.03; 16.60]	0.644^d^
Modifiable risk factors for dementia				
Hypertension, n (%)	114 (90.5)	43 (89.6)	71 (91)	0.789^c^
Diabetes mellitus, *n (%)*	33 (26.2)	9 (18.8)	24 (24.4)	0.136^c^
Years of education, *median* *[Q1; Q3]*	14.00 [12.00; 17.00]	13.00 [12.00; 16.00]	14.00 [12.00; 17.00]	0.348^d^
History of smoking, *n (%)*	39 (31)	10 (20.8)	29 (37.2)	0.054^c^
History of excessive alcohol consumption,* n (%)*	4 (3.2)	0 (0)	4 (5.1)	0.111^c^
Depression, *n (%)*	13 (10.3)	5 (10.4)	8 (10.3)	0.977^c^
Hyperlipidemia*, n (%)*	46 (36.5)	16 (33.3)	30 (38.5)	0.561^c^
Physical inactivity,* n (%)*	34 (27)	10 (20.8)	24 (30.8)	0.222^c^

*n* number of patients; *SD* standard deviation; *KTx* kidney transplant; *Q1* quartile 25’; *Q3* quartile 75’, ^*^p < 0.05, ^a^*One-sample Chi-squared test,* ^b^*Student-t test,* ^c^*Chi-Squared Χ *^*2*^*,* ^d^*U Mann–Whitney test*

The average age of the participants was 51.20 ± 12.67 years, with no significant differences observed among the groups. Besides the age distribution, dialysis vintage prior to KTx, and the interval from KTx to cognitive assessment were comparable between female and male cohorts. Moreover, no statistically significant differences in comorbid diseases were observed between the groups, as assessed by the Charlson Comorbidity Index. The prevalence of cognitive impairment was also similar between the groups, affecting 11 females and 18 males, respectively (*p* = 0.983). Biochemical analyses demonstrated higher serum creatinine levels in males than in females (1.57 vs. 1.25 mg/dL; *p* = 0.001), though creatinine clearance rates were similar between the groups (49.03 ± 20.29 vs. 50.79 ± 21.7 mL/min/1.73 m^2^; *p* = 0.645). Males also had higher urea levels (59.50 vs. 55.00 mg/dL; *p* = 0.046) and higher uric acid levels (7.20 vs. 6.20 mg/dL; *p* = 0.012). Apart from a non-significant trend toward higher smoking prevalence among males (*p* = 0.054), there were no significant intergroup differences in modifiable risk factors.

### Cognitive screening results

The median time from KTx to cognitive assessment was 2.49 months, and the prevalence of cognitive impairment in the entire cohort was 23%. No statistically significant differences were observed between males and females, with prevalence rates of 23.1% and 22.9%, respectively. Table 2 shows major MACE scores.

**Table 2:** Mini-Addenbrooke’s Cognitive Examination results

	All participants *(n* = *126)*	Females *(n* = *48)*	Males *(n* = *78)*	*p*-value
MACE total score (/30) *mean* ± *SD* *median* *[Q1; Q3]*	26.75 ± 3.28 28.00 [26.00; 29.00]	27.10 ± 2.23 27.00 [26.00; 29.00]	26.54 ± 3.78 28.00 [26.00; 29.00]	0.917^a^
Attention (/4) *mean* ± *SD* *median* *[Q1; Q3]*	3.89 ± 0.36 4.00 [4.00; 4.00]	3.92 ± 0.28 4.00 [4.00; 4.00]	3.87 ± 0.41 4.00 [4.00; 4.00]	0.693^a^
Memory (/14) *mean* ± *SD* *median* *[Q1; Q3]*	12.48 ± 2.10 13.00 [12.00; 14.00]	12.79 ± 1.49 13.00 [12.00; 14.00]	12.29 ± 2.39 13.00 [12.00; 14.00]	0.464^a^
Semantic fluency (/7) *mean* ± *SD* *median* *[Q1; Q3]*	6.27 ± 0.95 7.00 [6.00; 7.00]	6.27 ± 0.98 7.00 [6.00; 7.00]	6.27 ± 0.94 7.00 [6.00; 7.00]	0.847^a^
Visuospatial abilities (/5) *mean* ± *SD* *median* *[Q1; Q3]*	4.19 ± 1.09 5.00 [5.00; 5.00]	4.17 ± 1.02 4.50 [3.25; 5.00]	4.21 ± 1.13 5.00 [3.75; 5.00]	0.578^a^

*n* number of patients; *SD* standard deviation; *Q1* quartile 25’; *Q3* quartile 75’; ^a^*U Mann–Whitney test*

The median MACE score was 28.00, with no significant differences observed between females and males (*p* = 0.917). No statistically significant disparities were identified across specific cognitive domains. Females scored slightly higher in the attention (3.92 ± 0.28 vs. 3.87 ± 0.41; *p* = 0.693) and memory (12.79 ± 1.49 vs. 12.29 ± 2.39; *p* = 0.464) domains, whereas males had marginally higher scores in the visuospatial ability tests (4.21 ± 1.13 vs. 4.17 ± 1.02; *p* = 0.578). Semantic fluency scores were comparable (*p* = 0.847).

**Fig. 1:**
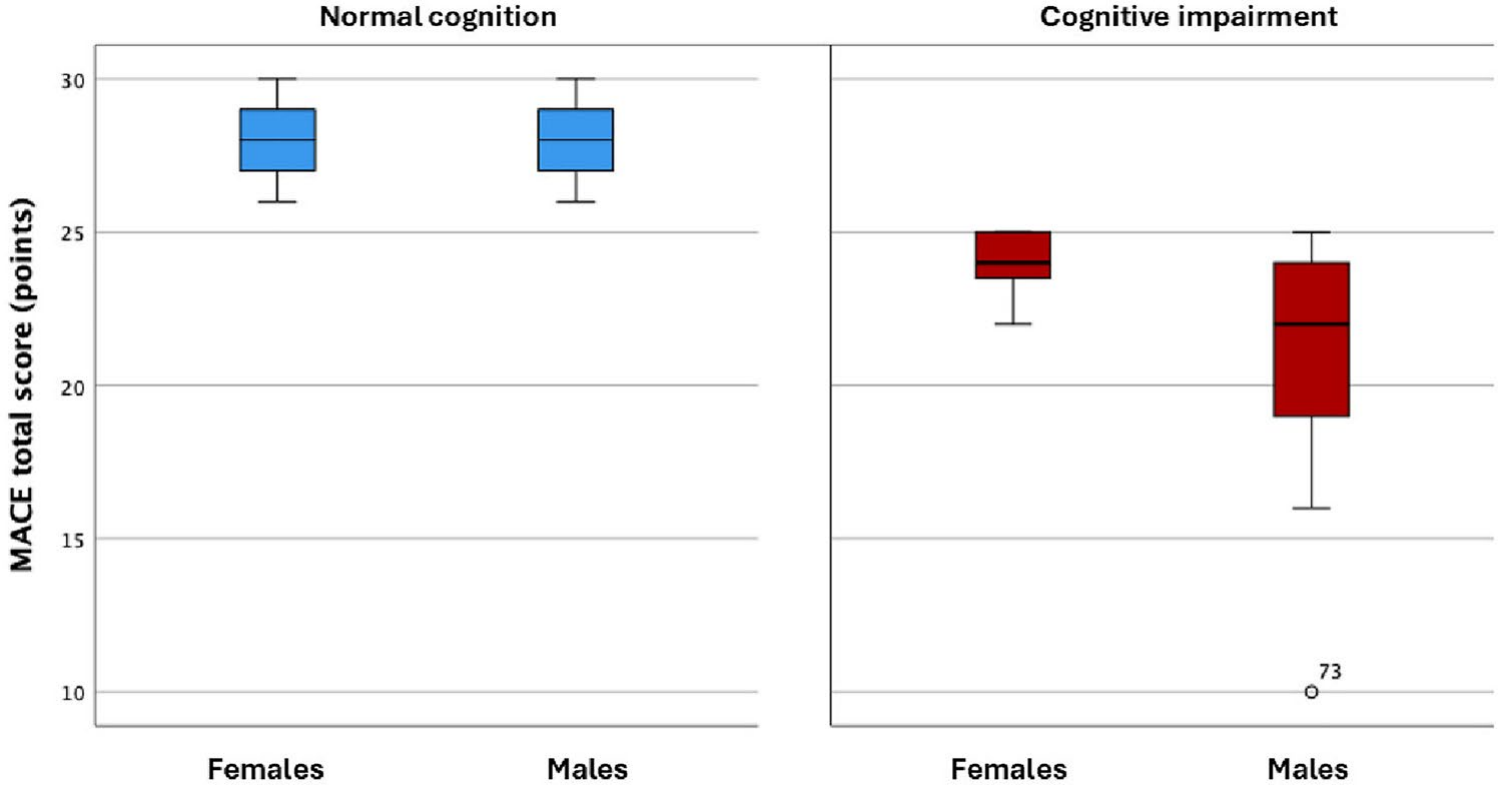
Total Mini-Addenbrooke’s Cognitive Examination (MACE) scores in females and males, divided by cognitive status: normal cognition (blue) and cognitive impairment (red)

Figure 1 presents MACE scores by cognitive status and sex. Participants with no cognitive impairment had similarly high scores across both sexes, reflecting preserved cognitive function. In contrast, individuals with cognitive impairment showed lower MACE scores. Females demonstrated a narrower interquartile range, while males showed a broader distribution. This suggests a greater variability and more pronounced impairment among men.

**Fig. 2:**
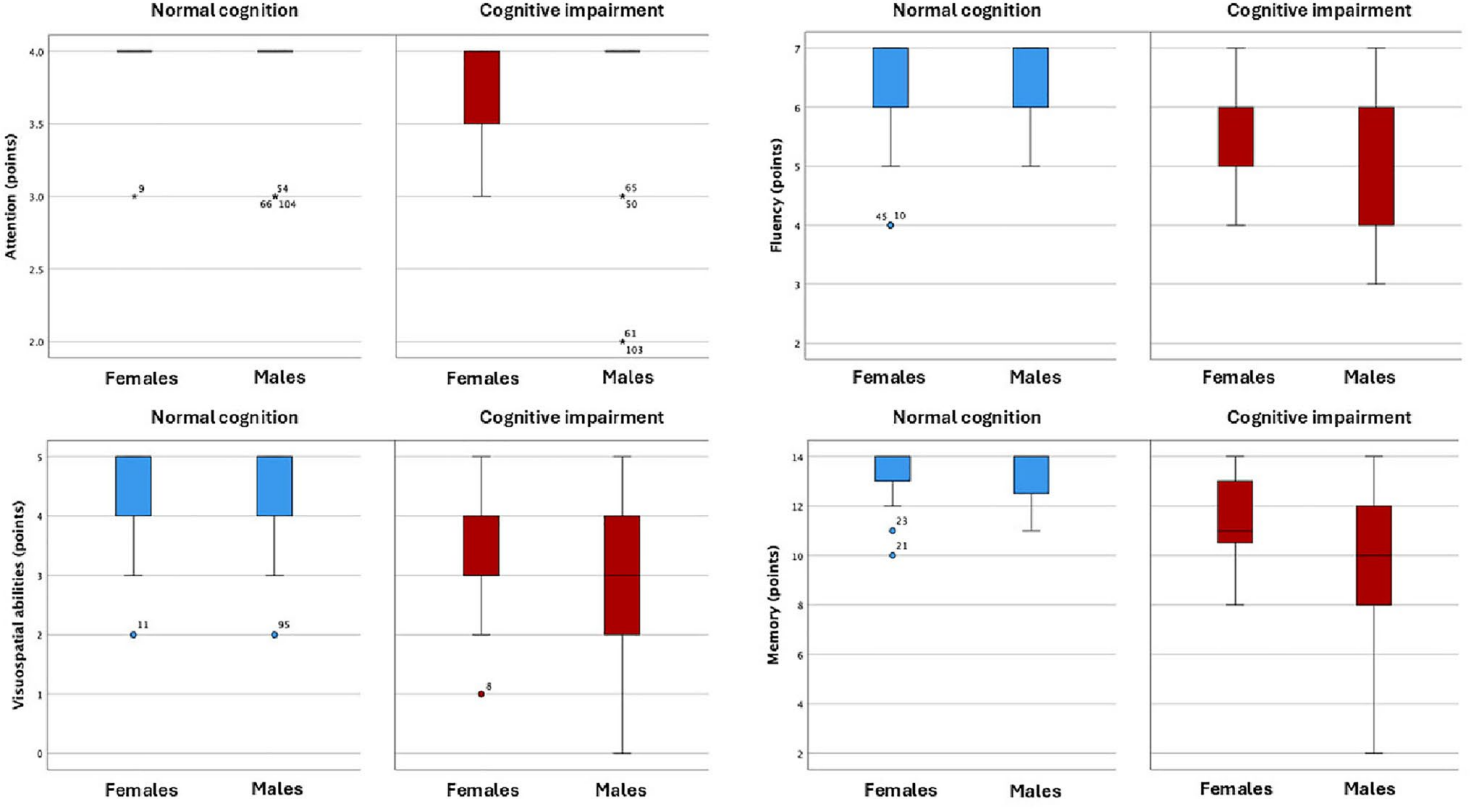
Mini-Addenbrooke’s Cognitive Examination (MACE) subscale scores in females and males, by cognitive status: normal cognition (blue) and cognitive impairment (red)

Figure 2 illustrates performance across various cognitive domains in male and female participants both with and without cognitive impairment, based on MACE subscale scores. Non-cognitive impairment participants demonstrated higher and more consistent scores in all domains—visuospatial abilities, fluency, attention, and memory—compared to the cognitive impairment group. Attention scores remained uniformly high in the non-cognitive impairment group. However, among participants with cognitive impairment, females exhibited greater variability in attention scores than males did. In contrast, males with cognitive impairment showed a broader distribution of scores across the remaining cognitive domains.

## Discussion

In this study, to the best of our knowledge for the first time in the literature, we investigated sex-based differences in cognitive impairment among transplant recipients. Overall, cognitive impairment affected 23% of the 126 study participants, with no significant sex-related difference in prevalence.

The reported prevalence of cognitive impairment among KTx recipients varies considerably across studies, ranging from 15.6% to 58% [8, 16], and remains higher than in the general population, even among younger recipients [16]. However, direct comparisons across studies are limited by substantial heterogeneity in cognitive screening instruments and methodological approaches, including the absence of established cut-off points and the lack of a healthy control group.

Based on assessments conducted prior to kidney transplantation and at 3 months and one year post-transplant, Gupta et al. demonstrated that kidney failure-related cognitive impairment may be partially reversible [17]. Their findings show that episodic and verbal declarative memory, as measured by Logical Memory I and II, returned to normative levels. However, semantic memory, verbal fluency, and language, as assessed by Category Fluency tasks for animal and vegetable naming, as well as psychomotor speed and visuospatial function, as measured by the Digit Symbol Substitution Test, showed only partial improvement. Although Mini-Mental State Examination scores improved slightly, they did not reach normative values [17]. Similarly, Binari et al. have reported improvements in attention and executive function, measured by the Trail Making Test, despite no significant changes in global cognition assessed with the Repeatable Battery for the Assessment of Neuropsychological Status [18]. Finally, studies evaluating cognitive performance in waitlisted patients with kidney failure have reported a high prevalence of cognitive impairment, affecting up to 30% of this population [19].

Thus, CKD is increasingly recognized as a risk factor for cognitive impairment, with vascular dementia representing the predominant underlying cause. However, the mechanisms involved are complex and poorly understood [6, 20]. Patients with CKD, particularly those with kidney failure, present with numerous cardiovascular risk factors commonly associated with vascular dementia pathogenesis, such as atherosclerosis, hypertension, atrial fibrillation, and diabetes mellitus. They are also exposed to CKD-specific factors, including uremic toxicity and chronic inflammation [20]. Furthermore, kidney transplant recipients who are treated with calcineurin inhibitors, such as tacrolimus or cyclosporine, may develop endothelial dysfunction and vasoconstriction, leading to reduced cerebral blood flow and subsequent cognitive impairment [6].

Assuming that vascular dementia is the main cause of kidney failure-related cognitive impairment, our findings support the concept that its overall prevalence does not differ significantly by sex [21, 22]. For instance, in a pooled analysis of four population-based studies involving individuals aged 65 years and older, Andersen et al. found that females over the age of 85 were at a higher risk of developing Alzheimer’s disease than males. However, no sex-related differences were observed in vascular dementia risk [21]. These results are further supported by an epidemiological study from the United Kingdom conducted by Imfeld et al., which demonstrated that vascular dementia affects males and females at comparable rates [22].

Moreover, our study population has a male predominance, which aligns with the common observation that females are less often referred and waitlisted for KTx, and less likely to receive a kidney transplant compared to males [23]. For instance, a national cohort study by Segev et al., involving 563,197 patients newly diagnosed with kidney failure between 2000 and 2005, found that females with comorbidities had lower access to KTx than males with the same conditions, despite similar survival benefits [24]. One potential explanation is that adult males are 50% more likely to develop kidney failure, despite the fact that CKD prevalence is higher in females [25]. Interestingly, a systematic review by Vilayur et al. revealed that females are more likely to serve as living donors, with female predominance observed in 83% of the studies [26]. Socioeconomic and emotional factors are among the most frequently mentioned explanations for sex disparities in living kidney donation. For instance, concerns about losing a primary source of income may discourage males from donating, while altruism may motivate females [26].

Additionally, although creatinine clearance rates were comparable for both sexes, in our study males had significantly higher creatinine levels than females. This finding is consistent with previous studies that emphasize the advantages of cystatin C as a marker of kidney function, particularly in individuals with greater muscle mass [27]. Furthermore, males in our study had significantly higher serum urea and uric acid levels compared to females, possibly because of the influence of sex hormones [28]. Wang and Charchar reported that circulating uric acid levels in adolescent boys were positively associated with higher testosterone levels and negatively associated with sex hormone-binding globulin concentrations [28]. Moreover, Liu et al. demonstrated blood urea nitrogen levels to be significantly higher in males than in females, regardless of age, which is consistent with our findings [29]. These results may be partially attributed to the lower muscle protein synthesis rates observed in males compared to females, as demonstrated by Henderson et al., this could result in increased protein breakdown and nitrogenous waste production [30].

This study has several limitations that should be acknowledged. First, the cross-sectional design prevents the establishment of causal relationships. Moreover, the small sample size, particularly in the female cohort, might have limited the statistical power to detect subtle differences. Furthermore, cognitive functions were assessed using the MACE rather than a comprehensive neuropsychological battery, which may limit diagnostic accuracy in the case of mild cognitive deficits [31]. Also, since some modifiable risk factors were self-reported by patients, recall bias may have compromised the accuracy of the collected data. Finally, the absence of a pre-transplant cognitive assessment hindered our ability to determine whether cognitive impairment developed de novo post-KTx or existed prior to transplantation. Despite these limitations, the study’s primary strength is in its novel focus on sex-related cognitive outcomes in kidney transplant recipients – an area that, to the best of our knowledge, has not yet been investigated.

## Conclusions

This study reveals a high prevalence of cognitive impairment among kidney transplant recipients, equally affecting males and females. The predominance of male participants in the cohort likely reflects systemic disparities in access to KTx. While graft function was similar between the groups, significant differences in creatinine, urea, and uric acid levels suggest sex-specific metabolic profiles, that may be influenced by hormonal factors and muscle mass. These findings highlight the importance of incorporating sex-specific considerations into the post-transplant care of KTx patients and of conducting powered, longitudinal studies with comprehensive cognitive testing to clarify the impact of sex on mechanisms and trajectories of cognitive impairment.

## Data Availability

All data generated or analyzed during this study are included in this published article.
